# Prognostic value of CDKN2A in head and neck squamous cell carcinoma via pathomics and machine learning

**DOI:** 10.1111/jcmm.18394

**Published:** 2024-05-15

**Authors:** Yandan Wang, Chaoqun Zhou, Tian Li, Junpeng Luo

**Affiliations:** ^1^ Department of Otolaryngology, Huaihe Hospital Henan University Kaifeng China; ^2^ Department of Pathology, Huaihe Hospital Henan University Kaifeng China; ^3^ School of Basic Medicine Fourth Military Medical University Xi'an China; ^4^ Translational Medical Center of Huaihe Hospital Henan University Kaifeng China

**Keywords:** cancer biomarkers, CDKN2A, digital pathology, HNSCC, machine learning in oncology

## Abstract

This study aims to enhance the prognosis prediction of Head and Neck Squamous Cell Carcinoma (HNSCC) by employing artificial intelligence (AI) to analyse CDKN2A gene expression from pathology images, directly correlating with patient outcomes. Our approach introduces a novel AI‐driven pathomics framework, delineating a more precise relationship between CDKN2A expression and survival rates compared to previous studies. Utilizing 475 HNSCC cases from the TCGA database, we stratified patients into high‐risk and low‐risk groups based on CDKN2A expression thresholds. Through pathomics analysis of 271 cases with available slides, we extracted 465 distinctive features to construct a Gradient Boosting Machine (GBM) model. This model was then employed to compute Pathomics scores (PS), predicting CDKN2A expression levels with validation for accuracy and pathway association analysis. Our study demonstrates a significant correlation between higher CDKN2A expression and improved median overall survival (66.73 months for high expression vs. 42.97 months for low expression, *p* = 0.013), establishing CDKN2A's prognostic value. The pathomic model exhibited exceptional predictive accuracy (training AUC: 0.806; validation AUC: 0.710) and identified a strong link between higher Pathomics scores and cell cycle activation pathways. Validation through tissue microarray corroborated the predictive capacity of our model. Confirming CDKN2A as a crucial prognostic marker in HNSCC, this study advances the existing literature by implementing an AI‐driven pathomics analysis for gene expression evaluation. This innovative methodology offers a cost‐efficient and non‐invasive alternative to traditional diagnostic procedures, potentially revolutionizing personalized medicine in oncology.

## 1 INTRODUCTION

Head and Neck Squamous Cell Carcinoma (HNSCC) is the most common cancer, responsible for around 90% of all tumours in the head and neck globally.^1^ Despite advancements in both surgical and adjuvant treatments, the overall prognosis for HNSCC patients remains discouraging, evidenced by a low 5‐year survival rate.^2^ The persistence of this dire prognosis underlines the pressing need for novel methodologies aimed at enhancing patient outcomes. In the age of precision medicine, traditional prognostic indicators for HNSCC, like clinical‐pathological characteristics and the status of Human Papillomavirus (HPV),^3^ are no longer satisfactory. A comprehensive investigation into the molecular basis of HNSCC has the potential to establish a structure for personalized and enhanced therapeutic treatments, with the potential involvement of the CDKN2A gene's expression in this regard.

Regulating cell cycle progression is a crucial function of the CDKN2A gene, which is well‐known for generating various transcripts that encode proteins such as p16 (INK4)^4^ and p14 (ARF).^5^ These proteins, by jointly regulating CDK4 and p53,^6^ supervise the transition from the G1 phase to the S phase.^7^ Mutations or deletions within this gene have been linked to numerous cancers, establishing its significance as a critical oncogene. Recent literature^8^, ^9^, ^10^ indicates that while apoptosis induction remains a common chemotherapeutic action mechanism, primary apoptosis regulators, notably p53 and p16/CDKN2A, are often inactivated within tumour tissues, which can lead to resistance against such treatments.

Despite its significance, the assessment of CDKN2A expression levels remains constrained by several limitations.^11^ Current detection methods, whether they utilize peripheral blood cytokine assays or are based on fresh or paraffin tissue specimens, are fraught with challenges related to cost, specimen collection, or reliability. There is a pressing need for methodologies that are both accurate and practical in clinical settings,^3^ especially given the fundamental role of CDKN2A in HNSCC prognosis.

The evolution of pathology has been marked by the advent of artificial intelligence.^12^ Pathomics,^13^ leveraging AI, has the potential to transform high‐quality, large‐scale data from pathology images into actionable insights. The application of Artificial Intelligence (AI) in cancer pathology is transforming the diagnostic landscape, offering unprecedented precision in analysing complex pathological data.^14^, ^15^ In particular, AI's role in enhancing gene expression analysis and its correlation with clinical outcomes is rapidly evolving, setting a new benchmark for personalized cancer care. This encompasses extracting texture and morphological features, edge gradient features, and even biological properties. The assessment of CDKN2A expression in HNSCC presents significant challenges, primarily due to the heterogeneity of tumour tissues and the limitations of traditional detection methods, which often require invasive biopsies and can yield variable results due to differences in technique sensitivity and specificity. Our proposed AI‐driven pathomics approach seeks to address these challenges by leveraging advanced machine learning algorithms to analyse pathologic images, offering a non‐invasive, highly accurate, and reproducible method for assessing CDKN2A expression. This innovative approach not only overcomes the limitations of sample heterogeneity and invasiveness but also provides a comprehensive analysis of tumour microenvironment features that are not discernible through conventional methods.

## 2 MATERIALS AND METHODS

### 2.1 Sample collection and preparation

**FIGURE 1:**
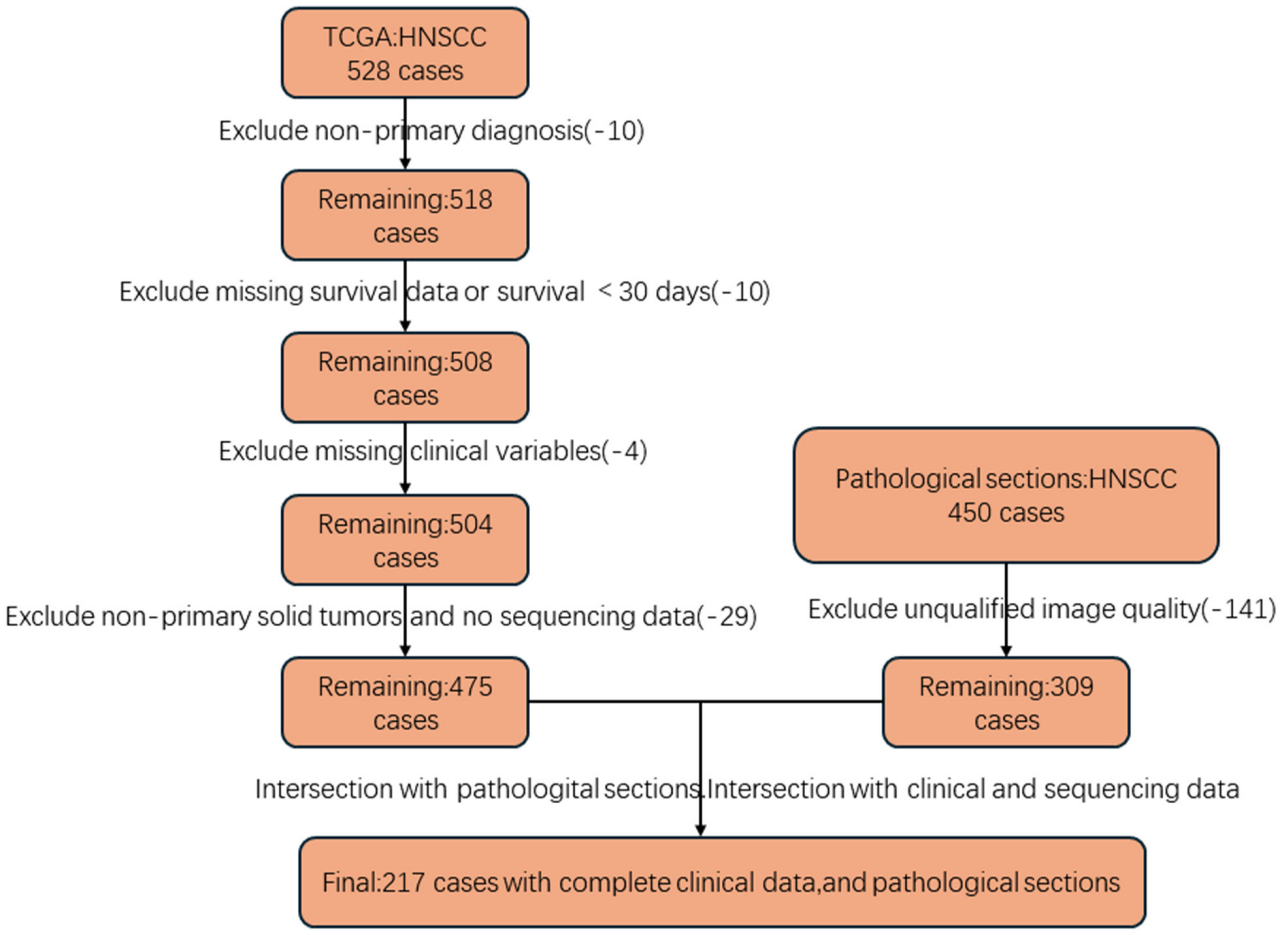
Head and Neck Squamous Cell Carcinoma case selection and data filtering process. The intersection of the 475 cases from the TCGA cohort with the 309 cases from the pathological sections yielded a final set of 271 HNSCC cases with complete clinical data, sequencing data, and pathological sections.

We selected 528 HNSCC cases from TCGA database. After excluding 53 cases, 475 cases with comprehensive clinical and transcriptomic data were included (Figure 1). To clarify, the 53 cases were excluded based on specific criteria, including insufficient tissue quality, lack of complete clinical data, and cases where CDKN2A expression could not be reliably assessed due to technical issues in the preparation process.

### 2.2 Prognostic analysis of CDKN2A expression

To determine the CDKN2A expression threshold for classifying patients into high‐risk and low‐risk groups, we utilized ROC analysis to identify the optimal point that maximized the Youden index, balancing sensitivity and specificity. This threshold was further validated through survival analysis, confirming its prognostic significance. Patients were categorized according to a CDKN2A expression threshold of 1.743. Table 1 shows the formation of groups with both high and low expression. Fragments Per Kilobase Million (FPKM) data underwent log2 transformation after being converted to Transcripts Per Million (TPM).^13^ Survival differences were visualized via Kaplan–Meier survival curves,^16^ with median survival times indicating 50% survival. Log‐rank tests were utilized for comparisons. The univariate and multivariate Cox regression analyses were used to evaluate the factors that impact overall survival (OS). Hazard Ratios (HR) were interpreted with values >1 indicating increased risk, and values <1 indicating protection.^17^

**TABLE 1:** Clinical characteristics of patients based on CDKN2A expression levels.

Variables	Total (*n* = 475)	Low (*n* = 208)	High (*n* = 267)	*p*
Gender, *n* (%)				0.031
Female	126 (27)	66 (32)	60 (22)	
Male	349 (73)	142 (68)	207 (78)	
Age, *n* (%)				0.979
<60	207 (44)	90 (43)	117 (44)	
≥60	268 (56)	118 (57)	150 (56)	
HPV_status, *n* (%)				<0.001
Negative	68 (14)	34 (16)	34 (13)	
Positive	30 (6)	2 (1)	28 (10)	
Unknown	377 (79)	172 (83)	205 (77)	
Systematic _therapy, *n* (%)				0.209
NO	313 (66)	144 (69)	169 (63)	
YES	162 (34)	64 (31)	98 (37)	
Radiotherapy, *n* (%)				0.941
NO	219 (46)	95 (46)	124 (46)	
YES	256 (54)	113 (54)	143 (54)	
Pathologic_stage, *n* (%)				0.015
I/II	90 (19)	39 (19)	51 (19)	
Ш/IV	320 (67)	151 (73)	169 (63)	
Unknown	65 (14)	18 (9)	47 (18)	
Perineural invasion, *n* (%)				0.408
NO	180 (38)	74 (36)	106 (40)	
Unknown	139 (29)	59 (28)	80 (30)	
YES	156 (33)	75 (36)	81 (30)	
Margin_status, *n* (%)				0.349
Negative	326 (69)	145 (70)	181 (68)	
Positive	55 (12)	28 (13)	27 (10)	
Unknown	50 (11)	17 (8)	33 (12)	
Close	44 (9)	18 (9)	26 (10)	
Primary_tumour_site, *n* (%)				<0.001
Larynx	105 (22)	54 (26)	51 (19)	
Oral Cavity	293 (62)	135 (65)	158 (59)	
Oropharynx/Hypopharynx	77 (16)	19 (9)	58 (22)	
Primary_diagnosis, *n* (%)				0.039
NOS	404 (85)	172 (83)	232 (87)	
Keratinizing	51 (11)	30 (14)	21 (8)	
Others	20 (4)	6 (3)	14 (5)	
Histologic_grade, *n* (%)				0.038
G1/G2	346 (73)	162 (78)	184 (69)	
G3/G4/GX	129 (27)	46 (22)	83 (31)	

*Note*: The clinical profiles of patients categorized by CDKN2A expression into high and low groups. Information includes: Gender: Differences noted between groups (*p* = 0.031). HPV Status: Notable difference in distribution (*p* < 0.001). Pathologic Stage: Classification with significant group variation (*p* = 0.015). Primary Tumour Site: Significant differences between groups (*p* < 0.001). Primary Diagnosis: Categories presented, with a noted difference (*p* = 0.039). Histologic Grade: Grading with group differences (*p* = 0.038).

### 2.3 Pathomics analysis

#### 2.3.1 TCGA pathology cross‐sectional sample analysis

Patients were classified into high and low expression groups based on a CDKN2A expression threshold of 1.743. Out of these, a total of 271 samples contained both pathological images and comprehensive gene matrices.

#### 2.3.2 Image acquisition and processing

TCGA‐sourced images, formalin‐embedded sections in SVS format, were acquired at 20× or 40× magnification.^18^ To acquire tissue regions from pathological sections, we employed the OTSU algorithm (https://opencv.org/). This algorithm, which is an image binarization threshold technique, effectively separates the image into undesired background and the essential tissue areas required for analysis. We partitioned a 40x picture into numerous sub‐images of 1024 × 1024 pixels; partitioned a 20× picture into multiple sub‐images of 512 × 512 pixels, and then enhanced their resolution to 1024 × 1024 pixels. Afterwards, the pathologists examined the images and removed sub‐images that had low quality (such as those with contamination, blurry visuals, or blank areas exceeding 50%). For further analysis, randomly select 10 sub‐images from each pathological image.^19^

#### 2.3.3 Feature extraction

Using the PyRadiomics library (http://pyradiomics.readthedocs.io/en/latest/), we extracted original features (both first‐ and second‐order features) from the normalized sub‐image, as well as higher‐order features (Wavelet (LL, LH, HL, HH)). In total, 465 features were obtained. By analysing the mean values of the 10 sub‐images for each patient, the pathological histological characteristic of each sample was computed.^20^

#### 2.3.4 Dataset partitioning and feature filtering

Table‐2 shows that a 70–30 division was employed for the training and validation datasets. Pathomics feature values underwent z‐score standardization. The selection of features involved the utilization of both the minimum redundancy maximum relevance (mRMR) algorithm and the recursive feature elimination (RFE) algorithm (Appendix S1).

#### 2.3.5 Gradient boosting machine (GBM) model construction and evaluation

**FIGURE 2:**
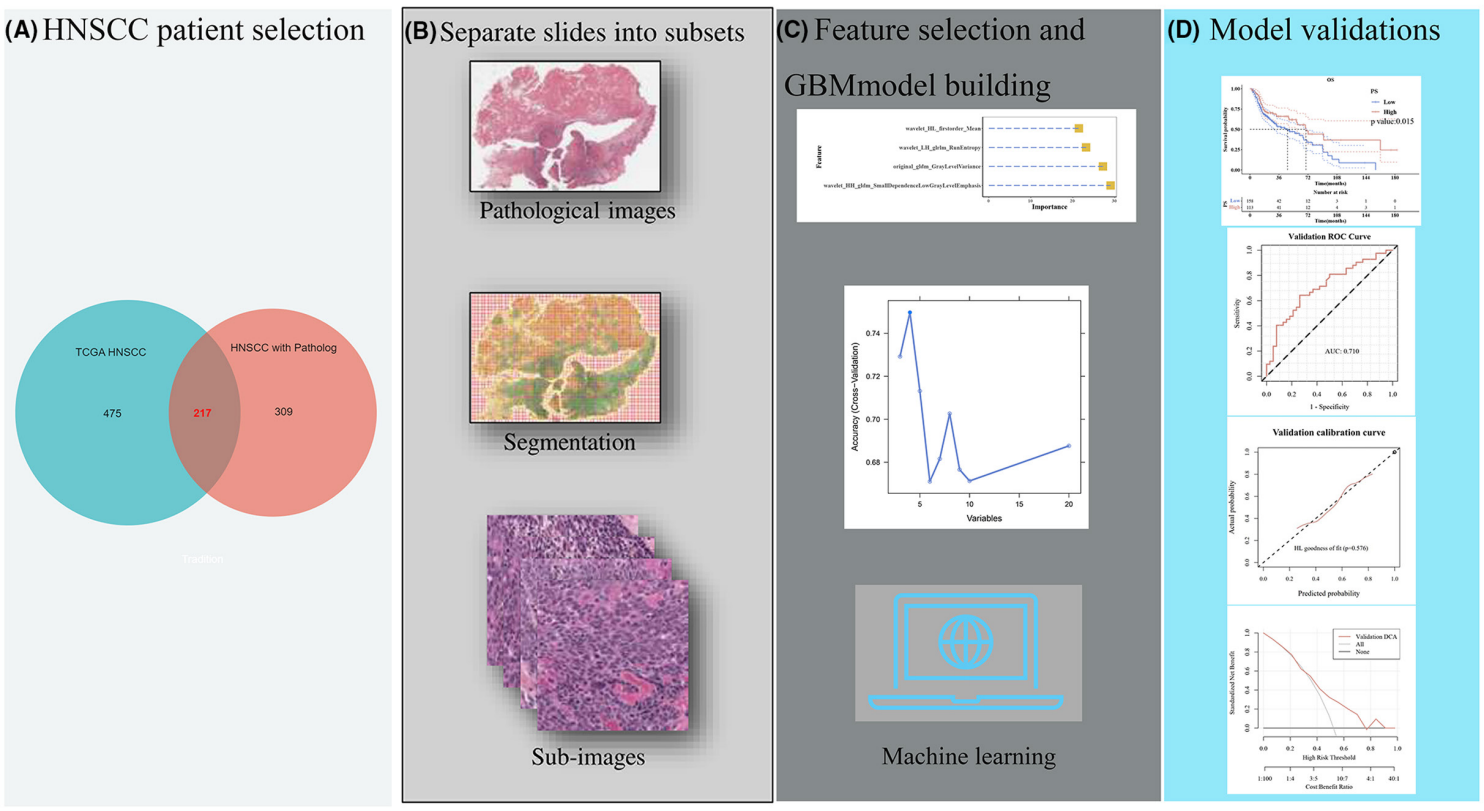
Workflow of HNSCC patient data analysis: from selection to model validation. (A) HNSCC patient selection: The left circle represents the TCGA HNSCC cohort, while the right circle denotes the HNSCC patients with pathological data. The overlapping region indicates the shared patients between the two cohorts, totaling 271. (B) Separate slides into subsets: Pathological images, Segmentation and Sub‐images. (C) Feature selection and GBM model building. (D) Model validations.

We employed the GBM algorithm on the shortlisted features for gene expression prediction.^21^ Multiple performance metrics were assessed, encompassing Accuracy (ACC), Specificity (SPE), Sensitivity (SEN), Positive Prediction Value (PPV), Negative Prediction Value (NPV), Receiver Operating Characteristic‐Area Under Curve (ROC‐AUC), Precision‐Recall‐Area Under Curve (PR‐AUC), and Brier score (Figure 2). We divided our dataset into five folds, ensuring a balanced distribution of high‐risk and low‐risk cases across each fold. This approach allowed us to iteratively train the model on four folds and validate it on the remaining fold, a process repeated five times to cover the entire dataset. Such a strategy ensures that every data point is used for both training and validation, enhancing the robustness and generalizability of our model.^22^

### 2.4 Mechanistic insights from pathomics

The R package ‘clusterProfiler’ was utilized to conduct Gene Set Enrichment Analysis (GSEA) on the KEGG (c2.cp.kegg.v7.5.1.symbols.gmt) gene set and Hallmark gene set in order to explore the molecular mechanisms that contribute to the variations in expression between the high and low expression groups of the PS score.

The Wilcoxon rank sum test was used to conduct differential expression analysis of cell cycle‐related genes (https://www.kegg.jp/) between groups with high and low PS expression. Genes that had a *p*‐value less than 0.05 were chosen for visualization, and their outcomes were displayed through box plots, offering a visual depiction of the information.

Data on genetic changes for patients with TCGA‐HNSC were acquired from the TCGA Data Portal at https://portal.gdc.cancer.gov/. A total of 268 samples were included in the intersection between these samples and pathological histology data. The MAF file stored somatic variant data, and the R package maftools^23^ was utilized for analysing the mutation data. As part of the analysis, the visualization included the top 15 genes that underwent mutations in patients with HNSC.

### 2.5 Tissue microarray (TMA) validation for CDKN2A expression

In our study, the oral cancer tissue microarray (Shanghai Outdo Bio., Shanghai, China; Cat No: HOraC080PG01) underwent a series of preparatory steps. Initially, the array was deparaffinized using xylene, followed by a sequential rehydration process in decreasing concentrations of ethanol. To retrieve the antigen, we utilized high‐pressure processing in a citrate buffer with a pH of 6.0. Afterwards, a 10‐minute procedure was performed using 3% H_2_O_2_ to inhibit the activity of naturally occurring peroxidase.

Post‐blocking, the array was thoroughly washed thrice, each for 5 min, with PBS. Next, we added 50 μL of the primary antibody, Anti‐CDKN2A (Proteintech Group, Inc., Wuhan, China; Cat No 10883‐1‐AP, 1:1000 dilution), and left it to incubate overnight at 4°C. Afterwards, a phase of re‐warming at room temperature was conducted. Afterward, we introduced the second antibody and kept it incubating at a temperature of 37°C for a duration of 1 h.

For visualization, DAB staining was meticulously performed, which was observed under a microscope. This step was followed by counterstaining with haematoxylin. After the staining procedures, the array was dehydrated and mounted for preservation.

We employed the PANNORAMIC whole‐slide scanner to perform panoramic scanning of the tissue microarray for the analysis. From each spot, three random fields were selected for detailed analysis using the ImageJ software (NIH, USA). The main factor evaluated was the mean optical density (AOD), which was determined by dividing the total optical density value (IOD) by the positive pixel coverage area.

### 2.6 Statistical analysis

All statistical analyses were performed using R statistical software (Version 4.3.3). Survival functions were estimated employing the Kaplan–Meier method, with differences in survival rates assessed through the log‐rank test. The association between CDKN2A expression levels and clinical outcomes was analysed using Cox proportional hazards regression models, where adjustments were made for known confounders. The comparison of CDKN2A expression between tumour tissues and adjacent non‐tumour tissues was conducted using paired *t*‐tests. A *p*‐value of <0.05 was deemed statistically significant, and all tests were conducted on a two‐sided basis.

## 3 RESULTS

### 3.1 Detailed examination of patient baseline characteristics

Our in‐depth analysis encompassed 475 cases of Head and Neck Squamous Cell Carcinoma (HNSCC) from the TCGA database. We meticulously classified these patients into groups with high and low CDKN2A expression, utilizing a pre‐established expression threshold. This classification unveiled a stark contrast in median overall survival times between the groups: the high‐expression cohort exhibited a significantly longer survival of 66.73 months compared to 42.97 months for the low‐expression cohort, a finding that is statistically significant (*p* = 0.013) and suggests a pivotal role of CDKN2A expression in patient prognosis (Table 1). Additionally, this stratification revealed significant disparities in gender distribution, primary tumour sites, and histologic grades between groups, emphasizing the intricate relationship between CDKN2A expression and clinical outcomes.

### 3.2 In‐depth prognostic value analysis of CDKN2A expression

**FIGURE 3:**
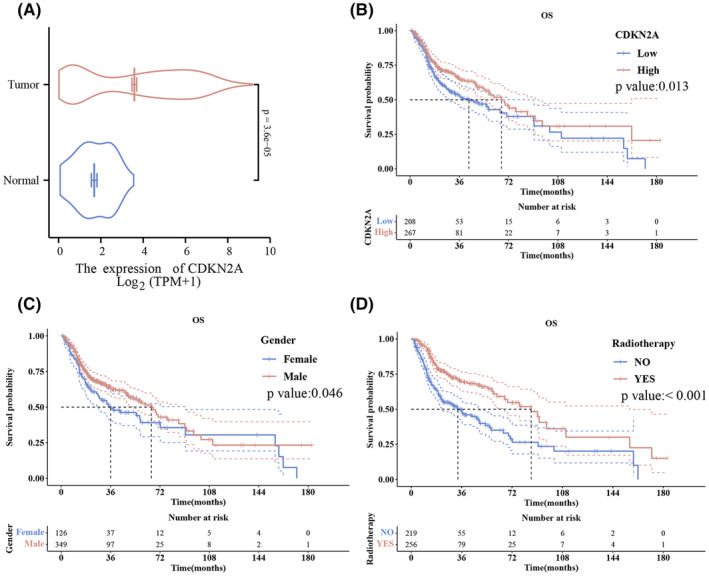
CDKN2A expression and survival outcomes based on gender and radiotherapy. (A) CDKN2A in Tumour vs. Normal: Violin plot showing CDKN2A expression levels in tumour (orange) and normal tissues (blue) on a Log2(TPM+1) scale. (B) Survival by CDKN2A Expression: Kaplan–Meier curves for high (red) vs. low (blue) CDKN2A expression, with *p*‐value: 0.013. (C) Survival by Gender: Kaplan–Meier curves for male (red) vs. female (blue) patients, *p*‐value: 0.046. (D) Survival & Radiotherapy: Kaplan–Meier curves for patients with (red) and without (blue) radiotherapy, *p*‐value: <0.001.

Further scrutinizing CDKN2A's prognostic significance, we discovered that higher levels of CDKN2A expression were robustly associated with enhanced overall survival outcomes (Refer to Figure 3). This association held true in both univariate (HR = 0.703, *p* = 0.014) and multivariate analyses (adjusted HR = 0.698, *p* = 0.019), solidifying CDKN2A's stance as an independent prognostic indicator. These findings underscore the gene's paramount importance in the oncological narrative of HNSCC, potentially serving as a beacon for therapeutic direction. Additional multivariate analysis provided further evidence supporting the protective effect of CDKN2A on OS (hazard ratio = 0.698, 95% confidence interval 0.517–0.943, *p*‐value = 0.019) (Appendix S1).

### 3.3 Baseline characteristics of TCGA pathology cross‐sectional samples

Upon evaluating the 271 TCGA pathology cross‐sectional samples, they were divided into training (191) and validation (80) sets based on CDKN2A expression (Table 2). Analysis of baseline characteristics, including age, gender, HPV status, and treatment history, showed no significant differences between the sets, affirming the comparability for our study's predictive analysis. This division ensures a balanced approach for the prognostic model's validation process (Table 3).

**TABLE 2:** Baseline characteristics comparison: training vs. validation sets.

Variables	Total (*n* = 271)	Train (*n* = 191)	Validation (*n* = 80)	*p*
CDKN2A, *n* (%)				1
Low	128 (47)	90 (47)	38 (48)	
High	143 (53)	101 (53)	42 (52)	
Gender, *n* (%)				0.432
Female	75 (28)	56 (29)	19 (24)	
Male	196 (72)	135 (71)	61 (76)	
Age, *n* (%)				0.639
<60	111 (41)	76 (40)	35 (44)	
≥60	160 (59)	115 (60)	45 (56)	
HPV_status, *n* (%)				0.321
Negative	44 (16)	34 (18)	10 (12)	
Positive	14 (5)	8 (4)	6 (8)	
Unknown	213 (79)	149 (78)	64 (80)	
Systematic_therapy, *n* (%)				0.052
NO	189 (70)	126 (66)	63 (79)	
YES	82 (30)	65 (34)	17 (21)	
Radiotherapy, *n* (%)				0.901
NO	132 (49)	94 (49)	38 (48)	
YES	139 (51)	97 (51)	42 (52)	
Pathologic_stage, *n* (%)				0.317
I/II	52 (19)	41 (21)	11 (14)	
III/IV	191 (70)	130 (68)	61 (76)	
Unknown	28 (10)	20 (10)	8 (10)	
Perineural_invasion, *n* (%)				0.806
NO	106 (39)	74 (39)	32 (40)	
Unknown	66 (24)	45 (24)	21 (26)	
YES	99 (37)	72 (38)	27 (34)	
Margin_status, *n* (%)				0.658
Close	36 (13)	26 (14)	10 (12)	
Negative	173 (64)	125 (65)	48 (60)	
Positive	39 (14)	26 (14)	13 (16)	
Uknown	23 (8)	14 (7)	9 (11)	
Primary_tumour_site, *n* (%)				0.349
Larynx	67 (25)	50 (26)	17 (21)	
Oral Cavity	171 (63)	121 (63)	50 (62)	
Oropharynx/Hypopharynx	33 (12)	20 (10)	13 (16)	
Primary_diagnosis, *n* (%)				0.157
Keratinizing	32 (12)	25 (13)	7 (9)	
NOS	230 (85)	162 (85)	68 (85)	
Others	9 (3)	4 (2)	5 (6)	
Histologic_grade, *n* (%)				0.595
G1/G2	204 (75)	146 (76)	58 (72)	
G3/G4/GX	67 (25)	45 (24)	22 (28)	
OS, *n* (%)				0.121
Alive	150 (55)	112 (59)	38 (48)	
Dead	121 (45)	79 (41)	42 (52)	
OS. Median (Q1, Q3)	22.1 (12.83, 44.47)	24.07 (13.12, 45.87)	18.37 (11.58, 39.19)	0.162

*Note*: There was no significant difference between the indicator groups, suggesting that the distributions of the training and validation sets were consistent.

**TABLE 3:** Clinical characteristics of patients categorized by PS score.

Variables	Total (*n* = 271)	Low (*n* = 158)	High (*n* = 113)	*p*
Gender, *n* (%)				0.625
Female	75 (28)	46 (29)	29 (26)	
Male	196 (72)	112 (71)	84 (74)	
Age, *n* (%)				0.844
<60	111 (41)	66 (42)	45 (40)	
≥60	160 (59)	92 (58)	68 (60)	
HPV_status, *n* (%)				0.136
Negative	44 (16)	29 (18)	15 (13)	
Positive	14 (5)	5 (3)	9 (8)	
Unknown	213 (79)	124 (78)	89 (79)	
Systematic_therapy, *n* (%)				0.155
NO	189 (70)	116 (73)	73 (65)	
YES	82 (30)	42 (27)	40 (35)	
Radiotherapy, *n* (%)				0.531
NO	132 (49)	80 (51)	52 (46)	
YES	139 (51)	78 (49)	61 (54)	
Pathologic_stage. *n* (%)				0.621
I/II	52 (19)	30 (19)	22 (19)	
III/IV	191 (70)	114 (72)	77 (68)	
Unknown	28 (10)	14 (9)	14 (12)	
Perineural invasion, *n* (%)				0.762
NO	106 (39)	61 (39)	45 (40)	
Unknown	66 (24)	41 (26)	25 (22)	
YES	99 (37)	56 (35)	43 (38)	
Margin_status, *n* (%)				0.459
Negative	173 (64)	107 (68)	66 (58)	
Close	36 (13)	18 (11)	18 (16)	
Positive	39 (14)	21 (13)	18 (16)	
Unknown	23 (8)	12 (8)	11 (10)	
Primary_tumour_site, *n* (%)				0.907
Larynx	67 (25)	40 (25)	27 (24)	
Oral Cavity	171 (63)	98 (62)	73 (65)	
Oropharynx/Hypopharynx	33 (12)	20 (13)	13 (12)	
Primary_diagnosis, *n* (%)				0.784
NOS	230 (85)	136 (86)	94 (83)	
Keratinizing	32 (12)	17 (11)	15 (13)	
Others	9 (3)	5 (3)	4 (4)	
Histologic_grade. *n* (%)				0.205
G1/G2	204 (75)	114 (72)	90 (80)	
G3/GA/GX	67 (25)	44 (28)	23 (20)	

*Note*: A breakdown of clinical information for patients segregated into high (*n* = 113) and low (*n* = 158) groups based on their PS scores. There was no significant difference between the indicator groups, indicating that the distribution of the PS score high and low groups at baseline was consistent.

### 3.4 Performance of the pathogenic model

**FIGURE 4:**
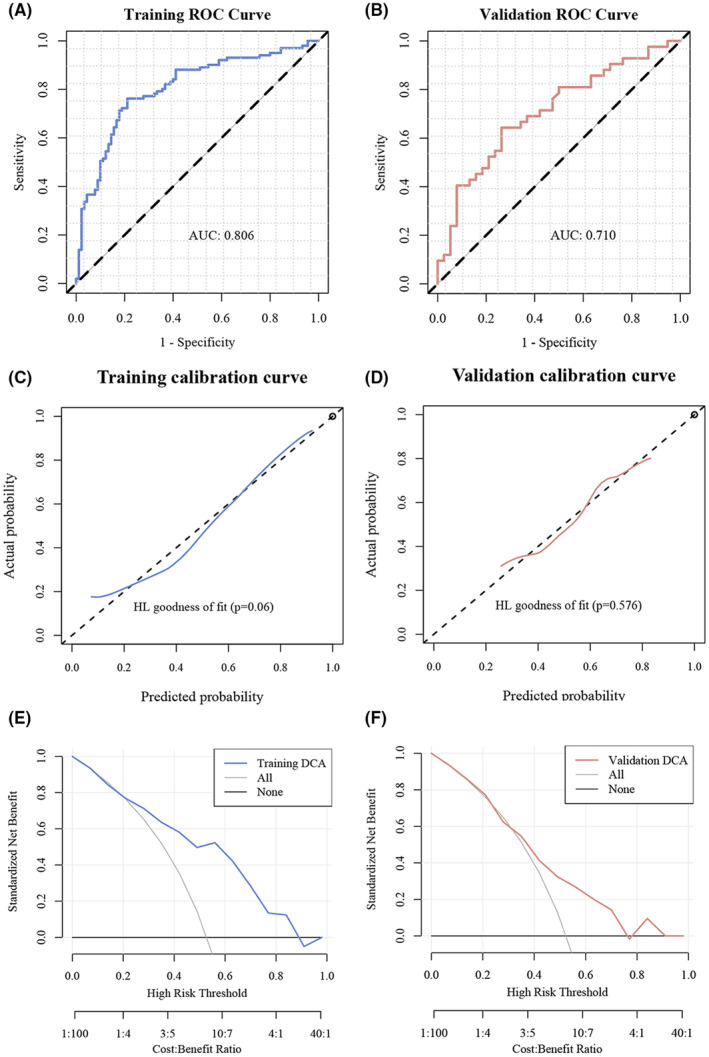
Evaluation of Predictive model performance. (A) Training ROC Curve. The plot showcases the Receiver Operating Characteristic (ROC) curve for the training dataset. The area under the curve (AUC) is 0.806, suggesting a good discriminative ability of the model for the training set. (B) Validation ROC Curve. This graph displays the ROC curve for the validation dataset. With an AUC of 0.710, it indicates a fair performance of the model on the validation set. (C) Training Calibration Curve. The calibration curve for the training dataset is depicted. The Hosmer‐Lemeshow (HL) goodness of fit test yields a *p*‐value of 0.06, suggesting a decent fit of the predicted probabilities to the actual outcomes. (D) Validation Calibration Curve. This is the calibration curve for the validation dataset. The HL goodness of fit test *p*‐value is 0.576, showing that the predicted probabilities are well‐calibrated with the actual results on the validation set. (E) Decision Curve Analysis (Training). The standardized net benefit is plotted against different risk thresholds for the training dataset. The curves represent different clinical decisions: treating all, treating none, or treating based on the model's prediction (Training DCA). The model's performance is assessed against different cost–benefit ratios, as indicated on the *X*‐axis. (F) Decision Curve Analysis (Validation). Standardized net benefit versus risk thresholds for the validation dataset is shown. Similar to the training DCA, the graph indicates the benefits of different clinical decisions at various cost–benefit ratios.

113 were classified into the high group, reflecting a higher expression of CDKN2A or features associated with a better prognosis, and 158 were classified into the low group, indicating lower expression or features associated with a poorer prognosis. The pathomic model showcases promising predictive capabilities with AUCs of 0.806 for the training set and 0.710 for the validation set, as depicted in Figure 4. Calibration curves and Hosmer‐Lemeshow tests confirm model alignment with actual outcomes (*p* > 0.05), while Decision Curve Analysis underscores its clinical utility.

**FIGURE 5:**
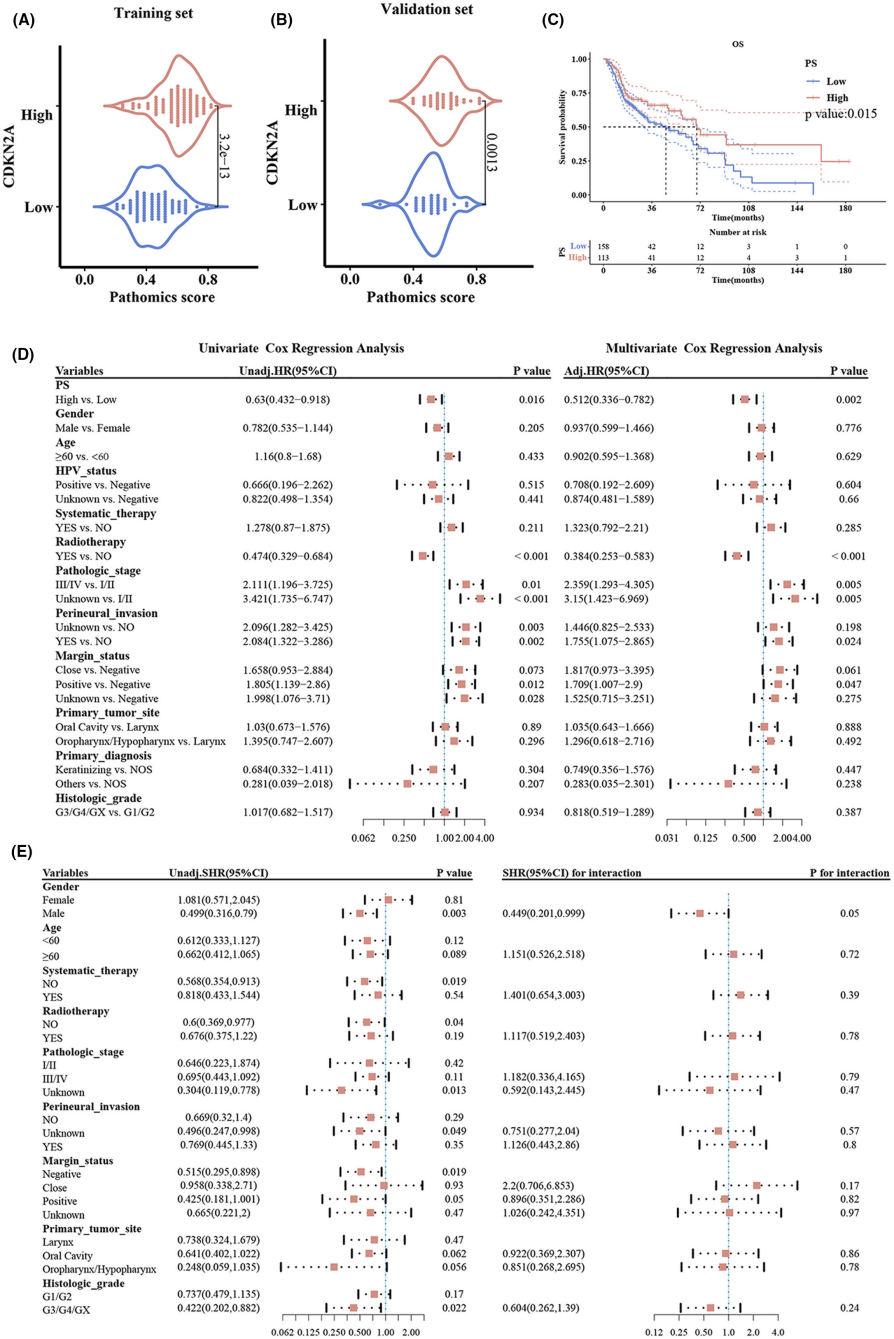
Comparative analysis of pathomics scores and prognostic factors. (A) Training set Pathomics Distribution: Violin plot showcasing the distribution of pathomics scores. Stratified by low and high CDKN2A expression. (B) Validation set Pathomics Distribution: Violin plot presenting the distribution of pathomics scores for the validation set, differentiated by low and high CDKN2A expression. The PS score in the validation set reflects the same expression of CDKN2A as in the training set. (C) Kaplan–Meier Survival Analysis: Survival probability over time for patients stratified by low and high PS. A significant difference is observed with *p*‐value: 0.015. (D) Prognostic Factors and Survival Analysis: Left: Univariate Cox Regression Analysis presenting Hazard Ratios (HR) for various prognostic variables. Right: Multivariate Cox Regression Analysis showcasing adjusted HRs. PS score is an independent risk factor. (E) Interaction Analysis between Variables: Univariate analysis presenting Subdistribution Hazard Ratios (SHR) for various variables. Right panel displays the SHR for interaction among these variables.

Distinct Pathomics scores, highlighted in Figure 5A,B, show clear differentiation between high and low CDKN2A expression groups. The GBM model identified a Pathomics score threshold of 0.5741 for risk categorization (Figure 5C), dividing patients into high (score of 113) and low (score of 158) risk groups. Kaplan–Meier analysis (*p* = 0.015) and both univariate and multivariate analyses confirmed high PS as a protective factor for overall survival.

Subgroup analysis, illustrated in Figure 5D,E, reveals gender‐specific associations, with elevated PS significantly benefiting males over females, without notable interaction effects between PS and gender.

### 3.5 Gene expression and tissue microarray validation insights

**FIGURE 6:**
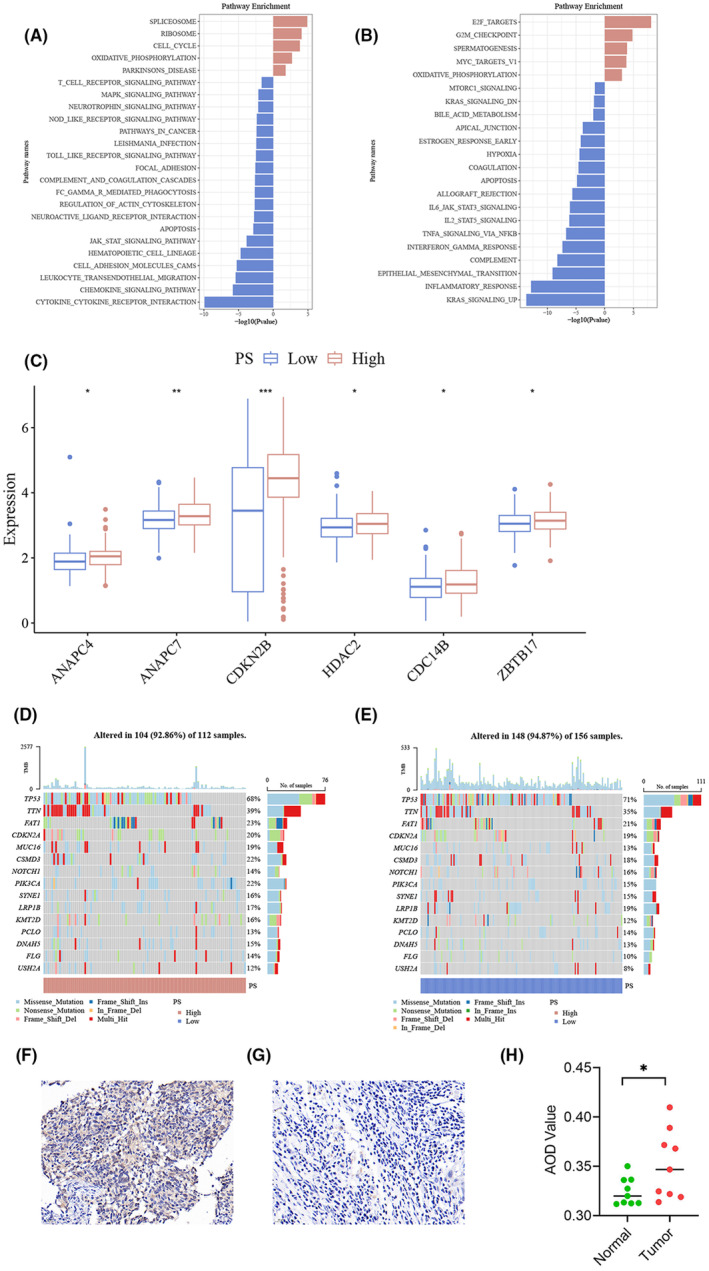
Molecular profiling and pathway enrichment analysis in tumour samples. (A) Pathway Enrichment Analysis based on KEGG gene set: Bar chart showcasing the top enriched pathways based on molecular alterations. The length of bars represents the −log10(*p*‐value) of enrichment, with red bars denoting enrichment in up‐regulated genes and blue bars in down‐regulated genes. (B) Pathway Enrichment Analysis based on Hallmark gene set: Another set of enriched pathways in a separate dataset or condition. The colour scheme and representation are similar to the left panel. (C) Differential analysis of cell cycle‐related genes: In the high and low PS expression groups, genes such as CDKN2B and NAPC7 show significantly higher expression in the high PS group compared to the low PS group (*p* < 0.01). (Significance markers: *p < 0.05; **p < 0.01; ***p < 0.001). (D) Genomic Alterations in Tumour Samples in high PS score An oncoprint highlighting the genomic alterations in 112 samples. Genes are listed vertically with alteration percentages. Different colours represent different types of genetic mutations. (E) Genomic Alterations in Tumour Samples in low PS scoreAn oncoprint for 156 samples. Genes and their alteration types are showcased similarly to the left panel. (F) Immunohistochemical expression in a case of oral squamous cell carcinoma tumour tissue (400× magnification). (G) Immunohistochemical expression in adjacent non‐tumour tissue of the same case (400× magnification). (H) Comparison of AOD values between cancer tissues and adjacent non‐tumour tissues in nine paired cases of oral squamous cell carcinoma. The AOD values were higher in cancer tissues compared to adjacent non‐tumour tissues, with a significance level of *p* = 0.0177.

Gene expression analysis and subsequent tissue microarray validation have elucidated distinct molecular pathways and confirmed CDKN2A's prognostic value in HNSCC. Our findings, represented in Figure 6A–H, reveal significant pathway enrichment differences between high and low PS expression groups. High PS expression correlates with cell cycle and G2M checkpoint activation (Figure 6A,B), highlighting a proliferation signature, whereas low PS expression is associated with JAK/STAT signalling and Epithelial‐Mesenchymal Transition (EMT), suggesting varied oncogenic activities. CDKN2B showed markedly higher expression in the high PS group, emphasizing the importance of cell cycle regulation in patient prognosis (Figure 6C). Tissue microarray validation further substantiated CDKN2A's role, aligning with our molecular findings. Figure 6F,G illustrate the differential CDKN2A expression in cancerous versus adjacent non‐tumour tissues, with Figure 6H quantitatively confirming this variation. Such validation not only reinforces CDKN2A as a critical prognostic marker but also underscores its potential in refining prognostic categorization within HNSCC.

## 4 DISCUSSION

### 4.1 Main interpretation

This study underscores a significant advancement in understanding HNSCC by elucidating CDKN2A's prognostic role through an AI‐driven pathomics approach. The observed disparity in median survival times between high‐expression and low‐expression cohorts not only highlights CDKN2A's prognostic relevance but also showcases the potential clinical usefulness of the GBM model, as reflected by its predictive effectiveness with an AUC of 0.806 in the training set. Furthermore, the GSEA's revelation of a strong association between high PS scores and pivotal cell cycle pathways enriches our molecular insight into HNSCC.

Building upon the foundational work in the field, our findings echo and extend the narrative around CDKN2A's prognostic significance in cancer.^24^, ^25^, ^26^ For instance, Swati et al.'s^27^ investigation into the relationship between CDKN2A/p16 expression and recurrence in Indian oral squamous cell carcinoma aligns with our insights, underlining the gene's broader applicability as a biomarker. Likewise, research by Madelene et al.,^28^ utilizing 5‐aza‐2'deoxycytidine (5‐aza‐dC) to reactivate p16 in Tu159 HNSCC cells, resulting in inhibited tumour growth, supports the functional implications of CDKN2A expression we observed. Furthermore, the study by William et al., which associates CDKN2A copy number loss with decreased survival rates, corroborates the clinical relevance of our findings.^29^ Similar to Xing's use of machine learning to identify prognostic biomarkers from pseudogenes,^30^ our study employs AI to analyse CDKN2A expression, enhancing prognostic accuracy in HNSCC.

Our approach, leveraging AI to correlate gene expression with pathological histological characteristics, offers an innovative perspective that complements Ali et al.'s methodology for utilizing H&E‐stained pathological slides for cellular nuclear spatial distribution analysis.^31^ This novel integration highlights the transformative potential of AI in pathomics, providing a more intuitive and effective tool for clinicians. The innovative application of artificial intelligence in pathomics within this study marks a transformative step forward in predicting CDKN2A expression, serving as a cost‐effective and minimally invasive alternative to conventional methodologies. The development of a pathomics‐based prognostic model has the potential not only to streamline clinical decision‐making processes but also to pave the way for highly personalized treatment strategies. Furthermore, this research significantly deepens our understanding of the molecular underpinnings of HNSCC. Notably, as depicted in Figure 6, there is an evident enhancement in cell cycle signalling pathways among genes with differential expression and high PS scores. This differentiation between high and low PS score groups underscores the possible tumour‐suppressive role of certain cell cycle‐related genes, like CDKN2B, aligning with existing literature that identifies CDKN2B as a tumour‐suppressor gene.^32^ It's also noteworthy that the high PS score group exhibited a lower incidence of TP53 mutations, although TP53 is renowned for its high mutation rate in HNSCC, often correlating with poorer prognoses.^33^

Neoplasms remain the main killer worldwide.^36^, ^37^, ^38^, ^39^, ^40^ The clinical relevance of our AI‐driven pathomics model is exceptionally promising. This model offers a non‐invasive and affordable means to assess CDKN2A expression, which could significantly contribute to the customization of oncology treatment plans. Integrating such a model into the clinical workflow has the potential to refine diagnostic processes, enhance decision‐making, and improve patient outcomes. To achieve these ends, implementing practical strategies to ensure the model's compatibility with existing healthcare IT infrastructures and providing concise training for medical professionals are paramount. Furthermore, engaging in discussions with regulatory bodies will be critical to navigating the model's clinical integration, ensuring adherence to data security and patient privacy regulations.^34^, ^35^

### 4.2 Limitations

Recognizing the limitations inherent in our retrospective design and the potential for selection bias, we echo the scientific community's call for prospective, multicentric studies that could further validate and refine the prognostic models based on CDKN2A expression. Such future endeavours, integrating broader omics data, promise not only to enhance the predictive accuracy of these models but also to deepen our understanding of the molecular intricacies of HNSCC, aligning with the concerted push towards precision medicine in oncology.

## 5 CONCLUSION

In conclusion, our research contributes significantly to the existing corpus of knowledge regarding CDKN2A's prognostic value in HNSCC, affirming the utility and promise of AI‐driven approaches in oncology. By bridging computational innovation with clinical application, our work not only underscores the pivotal role of CDKN2A in cancer prognosis but also marks a step forward in the quest for personalized cancer therapies, aligning with the broader objectives of precision medicine.

## AUTHOR CONTRIBUTIONS

**Yandan Wang:** Data curation (equal); formal analysis (equal); investigation (equal); methodology (equal); resources (equal); writing – original draft (equal). **Chaoqun Zhou:** Validation (equal); writing – review and editing (equal). **Tian Li:** Methodology (equal); resources (equal); writing – review and editing (equal). **Junpeng Luo:** Conceptualization (equal); funding acquisition (equal); project administration (equal); writing – original draft (equal); writing – review and editing (equal).

## FUNDING INFORMATION

Henan Provincial Medical Science and Technology Public Relations Program Provincial Ministerial Co‐Construction Key Project (SBGJ202302093) and Henan University Interdisciplinary Advanced Research Institute Construction Project (CX3070A0970005).

## CONFLICT OF INTEREST STATEMENT

The authors declare no competing interests.

## Supporting information


Appendix S1.

## Data Availability

Data are available on https://github.com/hicccp/CDKN2A.
