# Supplementary material for: Prognostic value of CDKN2A in head and neck squamous cell carcinoma via pathomics and machine learning
Source: J Cell Mol Med. 2024 May 15;28(9):e18394. doi: 10.1111/jcmm.18394 (PMC11096642; doi:10.1111/jcmm.18394)
Supplement: Supplementary file 1 — Appendix S1. [file JCMM-28-e18394-s001.pdf]

Supplementary materials

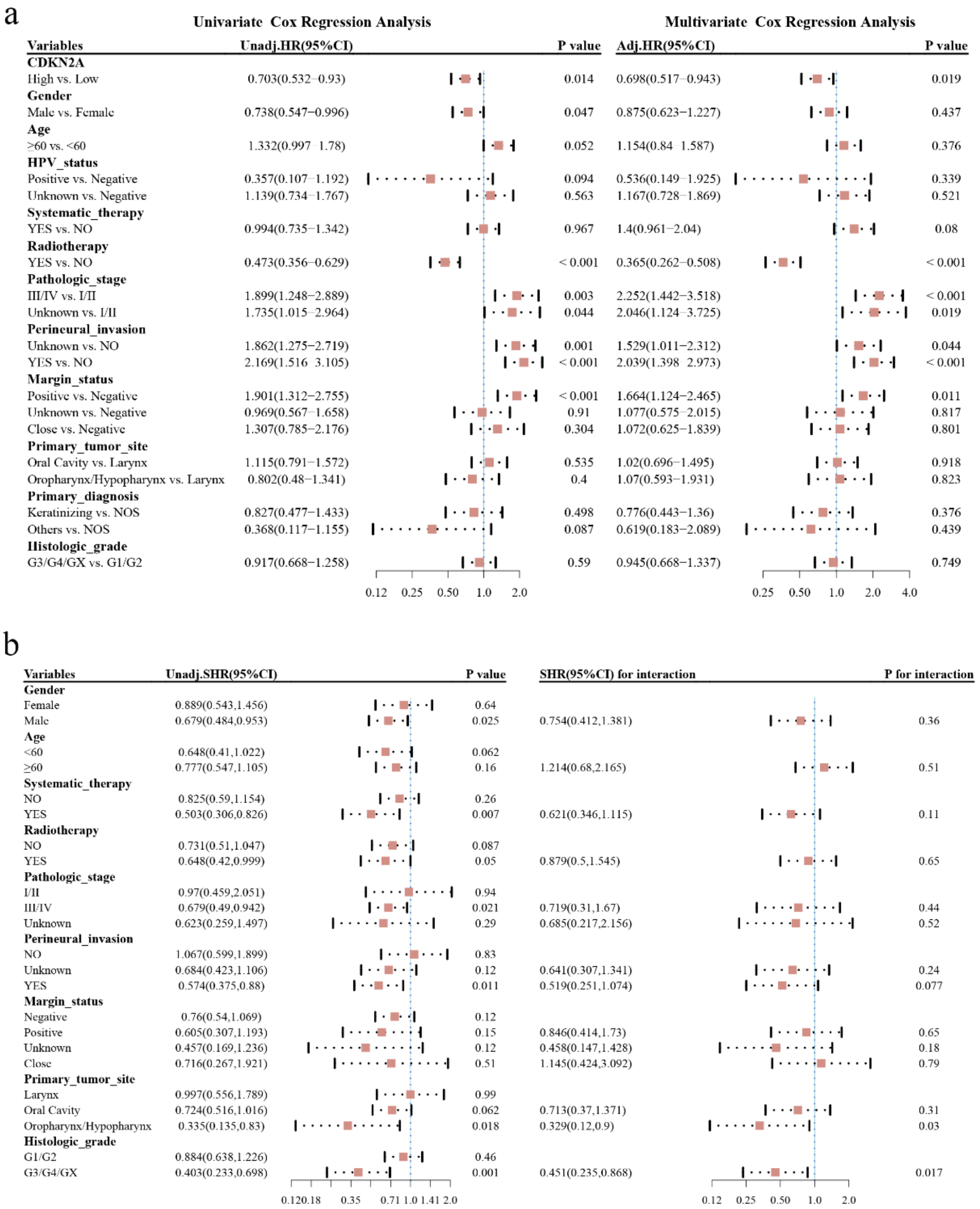

Fig-1 Comparative Analysis of CDKN2A and Prognostic Factors

In univariate analysis, high expression of CDKN2A was a protective factor for overall survival (OS) with statistical significance (HR=0.703, 95% CI 0.532-0.93, P=0.014). In multivariate analysis, after adjustment for multiple factors, high expression of CDKN2A (HR=0.698, 95% CI 0.517-0.943, P=0.019) remained a significant protective factor for OS. In subgroup analysis, for the female subgroup, elevated CDKN2A was a protective factor for OS (HR=0.889, 95% CI 0.543-1.456, P=0.64), although this was not statistically significant. Conversely, in the male subgroup, an increase in CDKN2A was also a protective factor for OS (HR=0.679, 95% CI 0.484-0.953, P=0.025), which was statistically significant. The interaction test yielded a P-value of 0.36, indicating no significant interaction between CDKN2A and the different gender subgroups, suggesting that the effect of CDKN2A on OS is consistent across gender subgroups.

Description about **mRMR_RFE** feature

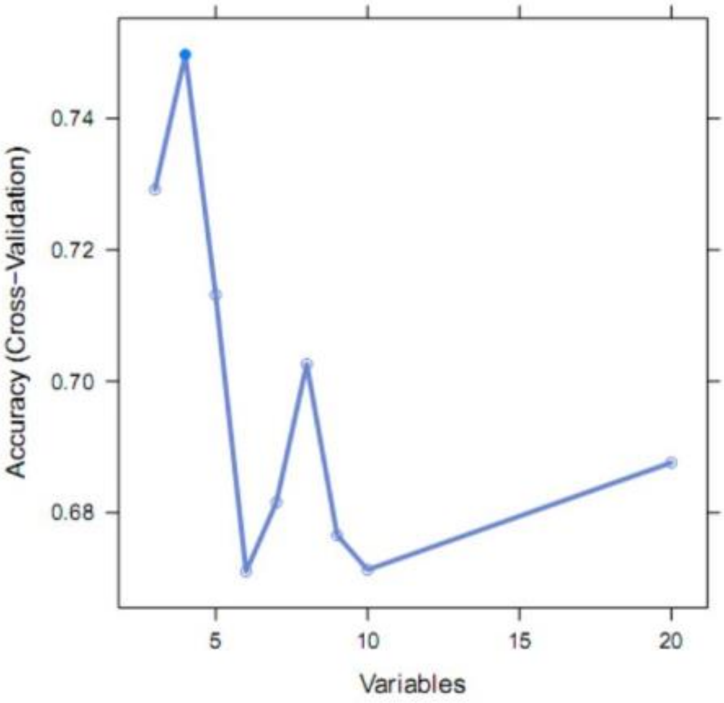

Fig-2 The mRMR method selected the top 20 features, and then RFE was used to select four features.

The mRMR algorithm (Maximum Relevance, Minimum Redundancy)[1] is used to select features, considering not only the correlation between features and the target variable to be predicted, but also the correlation between features themselves. The metric used is Mutual Information. For the mRMR method, the relevance of a feature subset to the class is calculated by the mean of the information gain of each feature with respect to the class, while the redundancy between features is measured by the sum of the mutual information between each feature and all other features in the subset, divided by the square of the number of features in the subset.RFE (Recursive Feature Elimination) [2]is a feature selection method that ranks the predictor variables before modeling and sequentially eliminates the least important ones. Its goal is to find the subset of predictors that can generate an accurate model. The model is repeatedly trained, with the n least important features being eliminated after each training. Then, the new feature subset is re-trained, and the feature importance is reassessed. The n least important features are again eliminated, until an optimal feature subset is obtained.

Description about built GBM Model

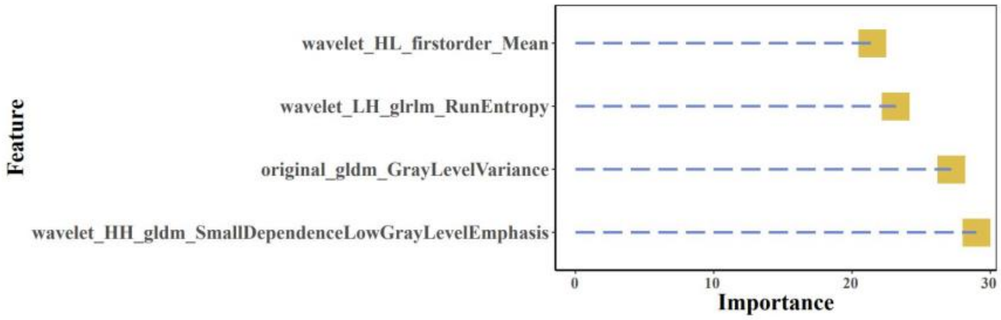

Fig-3 The importance of the selected features in the GBM algorithm.

Gradient Boosting Machine (GBM) algorithm[3] uses a set of weak classifiers (usually decision trees) to train new weak classifiers based on the negative gradient information of the current model's loss function. Then, the trained weak classifiers are combined with the existing model in an additive manner to build a predictive model. By using GBM to model the selected pathological features, it can predict gene expression.

Table-1 Comparison of parameters between training set and validation set

	ACC	SEN	SPE	Brier score
Training set	0.775	0.762	0.789	0.196
Validation set	0.650	0.690	0.605	0.223

PS: Accuracy (ACC), Specificity (SPE), Sensitivity (SEN)

References

[1] Zhang Y, Ding C, Li T. Gene selection algorithm by combining reliefF and mRMR. BMC GENOMICS. 2008. 9: 1-10.

[2] Granitto PM, Furlanello C, Biasioli F, Gasperi F. Recursive feature elimination with random forest for PTR-MS analysis of agroindustrial products. CHEMOMETRICS AND INTELLIGENT LABORATORY SYSTEMS. 2006. 83(2): 83-90.

[3] Ayyadevara VK, Ayyadevara VK. Gradient boosting machine. Pro machine learning algorithms: A hands-on approach to implementing algorithms in python and R. 2018 : 117-134.
